# Microsatellite instability due to hMLH1 deficiency is associated with increased cytotoxicity to irinotecan in human colorectal cancer cell lines

**DOI:** 10.1038/sj.bjc.6604691

**Published:** 2008-10-21

**Authors:** E Vilar, M Scaltriti, J Balmaña, C Saura, M Guzman, J Arribas, J Baselga, J Tabernero

**Affiliations:** 1 Department of Medical Oncology and Laboratory of Oncology Research, Vall d'Hebron University Hospital, Barcelona 08035, Spain

**Keywords:** colorectal cancer, microsatellite instability, *RAD50*, *MRE11*, irinotecan

## Abstract

Around 15% of colorectal cancers (CRCs) show microsatellite instability (MSI) due to dysfunction of the mismatch repair system (MMR). As a consequence of this, MSI tumours tend to accumulate errors in mononucleotide repeats as those in genes implicated in repairing double-strand breaks (DSBs). Previous studies have shown that irinotecan (CPT-11), a chemotherapy agent inducing DSB, is more active in MSI than in microsatellite stable (MSS) CRC. The purpose of this study was to compare the sensitivity to CPT-11 in a series of CRC cell lines with either proficient or deficient MMR and to assess the mutational status of two DSB repair genes, *MRE11* and *RAD50*, in these cell lines. *hMLH1*-deficient cell lines due to either epigenetic silencing or mutation showed very similar IC_50_ and were four- to nine-fold more sensitive to CPT-11 than the MSS line. Cell lines harbouring mutations in both *MRE11* and *RAD50* were most sensitive to CPT-11. We conclude that MSI cell lines display higher sensitivity to CPT-11 than MSS cells. Mutation of *MRE11* and *RAD50* could account for this difference in response to CPT-11. Future clinical trials tailoring chemotherapy regimens based on microsatellite status are warranted.

DNA mismatch repair (MMR) proteins correct three types of defects that escape the intrinsic proofreading exonuclease activity of DNA polymerases: (i) single base-pairing errors, (ii) unequal crossing over between microsatellites, and (iii) insertion/deletion loops that result from slippage during replication of repetitive sequences or during recombination. Microsatellites are multiple tandem repeats of a small number of nucleotides that are very prone to these errors; therefore MMR system activity is critical for their maintenance (Kunkel, 2004; Jiricny, 2006). On account of the fact that microsatellites are widely distributed in our genome, mutations of MMR genes affect multiple genetic targets, as those described in mononucleotide repeats of the DNA double-strand breaks (DSBs) repair genes *BLM*, *ATR*, *DNA-PK*, *BRCA2*, *RAD50*, and *MRE11*.

Colorectal cancers (CRCs) are classified as either displaying high-frequency microsatellite instability (MSI-H), low-frequency MSI (MSI-L), or microsatellite stability (MSS) depending on the number of microsatellite loci showing errors by previously defined consensus criteria (Giardiello *et al*, 2001). Around 15–20% of CRCs are MSI-H, mainly due to epigenetic silencing of the *hMLH1* gene promoter (Herman *et al*, 1998), whereas 2–3% of the total of CRCs are due to germ-line mutations in the MMR genes *hMLH1, hMSH2, hMSH6*, and *PMS2*, which are the cause of hereditary non-polyposis CRC (HNPCC) cases (Aaltonen *et al*, 1998; Salovaara *et al*, 2000). MSI-H sporadic tumours are characterised by high histologic tumour grade, right-sided location, young age of onset, lower pathological stage, mucinous phenotype with prominent tumour infiltrating lymphocytes, and better prognosis in terms of overall survival than MSI-L/MSS cases (Gryfe *et al*, 2000; Popat *et al*, 2005).

CPT-11 is a camptothecin analogue that binds reversibly to DNA topoisomerase I (TOP1) and traps it on the DNA strand, so cleavable complexes will remain stabilised and DNA DSBs will be generated after DNA or RNA polymerases collide with those complexes. This mechanism of action has been named as ‘the fork collision model’ (Pommier, 2006). MMR-deficient CRC tumours and cell lines frequently tend to accumulate mutations within microsatellite repeats of genes implicated in DSB repair pathway (eg, *MRE11* and *RAD50*) (Giannini *et al*, 2002; Koh *et al*, 2005), suggesting an enhanced sensitivity of these tumours to camptothecin analogues. In accordance with this fact, emerging clinical data suggest that MSI-H CRC patients may obtain more benefit from CPT-11-based chemotherapy than patients bearing MSS tumours (Fallik *et al*, 2003; Bertagnolli *et al*, 2006). Still, preclinical evidence suggesting a higher sensitivity of MMR-deficient tumours to irinotecan (CPT-11) is controversial due to discrepant results coming from different studies (Hausner *et al*, 1999; Jacob *et al*, 2001; Magrini *et al*, 2002; Fedier and Fink, 2004).

The objective of this study was to compare the sensitivity to CPT-11 in a series of CRC cell lines classified based on the microsatellite and the mutational status in coding mononucleotide repeats of *MRE11* and *RAD50*. Additionally, we aimed to assess the differences in sensitivity between cell lines with a genetic mutation in *MMR* genes (*MLH1* or *MSH6*), which resemble HNPCC, and cell lines with silencing of the *hMLH1* gene due to the promoter hypermethylation, such as sporadic MSI-H CRC cases.

## Materials and methods

### Cell lines and culture conditions

HCT-116, SW-48, RKO, and HCT-15 were kindly provided by Dr Manel Esteller (Cancer Epigenetics Laboratory, Spanish National Cancer Centre, Madrid, Spain). HT-29 was obtained from the American Type Culture Collection (Manassas, VA, USA). The microsatellite status of cell lines, the MMR gene mutational status and the analysis of the methylation of *hMLH1* promoter was ascertained from the literature and are summarised in Table 1 (Suter *et al*, 2003). Cells were maintained as monolayers at 37°C in 5% CO_2_ air in DMEM : Ham's F-12 containing 10% foetal bovine serum, glutamine (2 mM), and penicillin/streptomycin (50 IU ml^−1^).

### Western blotting

Cells were grown in 100-mm dishes until subconfluence. After removal of media, cells were washed twice with ice-cold PBS and scraped into ice-cold lysis buffer. After removal of cell debris by centrifugation, protein concentration was determined by Lowry assay (DC Protein assay, Bio-Rad, Hercules, CA, USA). Lysate samples containing equal amounts of protein were then added to SDS–PAGE loading buffer with 5% *β*-mercaptoethanol and heated 5 min at 100°C. Electrophoretic transfer to nitrocellulose membranes was followed by immunoblotting with the primary antibodies. Finally, membranes were hybridised with the appropriate horseradish peroxidase-conjugated secondary antibody (Amersham Pharmacia Biotech, Little Chalfont, UK), and were detected through chemiluminescence with the SuperSignal West Dura Extended Duration Substrate (Pierce, Rockford, IL, USA). Rabbit polyclonal antibodies against Mlh-1, Msh-2, and Msh-6 (Upstate, Dundee, UK) were used at 1 : 1000.

### Detection of mononucleotide repeats mutations of *MRE11* and *RAD50*

Mutation analyses for poly(T)11 nucleotide repeats in IVS-4 of *MRE11* and poly(A)9 nucleotide repeats in the ORF of *RAD50* were performed on genomic DNA extracted using the DNeasy kit (Qiagen, Valencia, CA, USA). PCR conditions and primers were set up as described previously (Kim *et al*, 2001; Giannini *et al*, 2002). A 3-*μ*l aliquot of the product was sequenced using the forward and reverse primers with an ABI BigDye TerV3.1 Cycle Sequencing kit on an ABI 9700 thermocycler (Applied Biosystems Inc., Foster City, CA, USA). The data were analysed using Sequencher 4.6 software (Gene Codes Corporation, Ann Arbor, MI, USA).

### Cytotoxicity assays and treatment of cultured cells

CPT-11 was obtained from Mayne Pharma Plc (Warwickshire, UK), and it was diluted in DMEM immediately before use. Drug cytotoxicity assays were performed using a modified tetrazolium dye colorimetric assay (cell proliferation reagent WST-1, Roche Applied Science, Penzberg, Germany). Viable cells will metabolise WST-1 to formazan by mitochondrial dehydrogenases and the quantification of formazan dye directly correlates with the number of metabolically active cells that was determined by a scanning microplate reader (ELISA reader). Three thousand cells were seeded per well in 96-well plates and were treated with increasing concentrations of CPT-11 for 48 h (1–100 nmol l^−1^) after 24 h. After that, cells were washed and incubated in drug-free medium for another 3 days. On day 6, WST-1 was added to each well and further incubated at 37°C for 1 h. Absorbances were measured and mean values of a minimum of six wells were calculated. Cultures in the absence of drugs were used as positive controls. The percentage of surviving cells at each concentration relative to the non-treated group was plotted and the drug concentrations resulting in 50% of growth inhibition (IC_50_) were calculated by linear regression analysis of the obtained dose–response curves.

### Cell cycle analysis

Cells were fixed with 70% ethanol at −20°C overnight, followed by treatment with 20 *μ*g ml^−1^ RNAse A and staining with propidium iodide. Fluorescence was measured on a Beckman coulter Epics® XL flow cytometer.

### Statistical analyses

Differences in CPT-11 sensitivity between cell lines displaying MSI or MSI-L/MSS were analysed using a two-sided *t*-test with *P*<0.05 considered statistically significant. Statistical analyses and linear regression for dose–response curves were performed using GraphPad Prism 4.0 (GraphPad Software, San Diego, CA, USA).

## Results

### Expression of MMR proteins and mutational status of *MRE11* and *RAD50*

**Figure 1:**
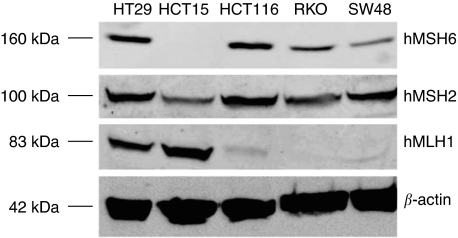
Expression of hMSH6 (160 kDa), hMSH2 (100 kDa), hMLH1 (83 kDa), and *β*-actin (42 kDa) were analysed by western blotting in whole-cell protein extracts.

Cell lines were selected to perform the experiments based on their microsatellite status. As shown in Table 1, four cell lines showed MSI. HCT-116 and HCT-15 presented biallelic mutations in *hMLH1*, *hMSH6* genes, respectively, resembling cases of patients affected by HNPCC. SW-48 and RKO had the *hMLH1* gene promoter silenced transcriptionally by hypermethylation as those sporadic tumours showing MSI-H. Contrary, HT-29 displayed normal MMR gene functioning as the majority of sporadic CRC cases. Expression of *hMLH1*, *hMSH2*, and *hMSH6* were ascertained by western blot (Figure 1). All CRC cell lines used in our study expressed some level of Msh2. The expression of Mlh1 was undetectable in HCT-116, SW-48, and RKO, whereas Msh6 protein was detected in all cell lines except for HCT-15, corresponding to the known mutational status.

We have studied the presence of mutations in microsatellite regions of *MRE11* and *RAD50* that are involved in surveillance and correction of DSBs, as those generated after treatment with CPT-11. In particular, we have investigated the occurrence of a frameshift mutation in a coding poly(A) repeat of *RAD50* and a shortening in an intronic poly(T) repeat of *MRE11*. These mutations result in a truncated protein in the case of *RAD50* and an aberrant transcript due to skipping of exon 5 and a premature stop codon introduction in *MRE11*. We showed that all MSI cell lines harboured mutations in the poly(T) tract of *MRE11* (Table 1). In addition, HCT-116 and RKO presented mutations in the poly(A) of *RAD50*. The MMR-proficient cell line HT-29 was wild type for both genes.

### Cytotoxicity of CPT-11 to CRC cell lines classified by microsatellite status

**Figure 2:**
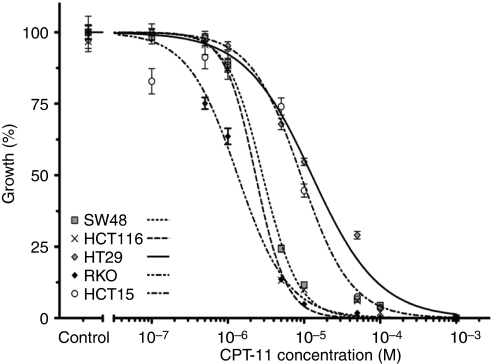
Cytotoxicity of CPT-11 to human colorectal cancer cell lines.

**Figure 3:**
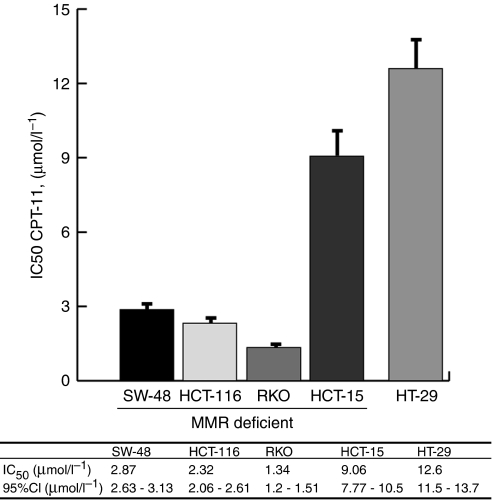
Cytotoxicity to CPT-11 of CRC cell lines. Histograms represent the IC_50_ and error bars represent 95% CI. IC50, concentrations resulting in 50% of growth inhibition; 95% CI, 95% confidence intervals.

Dose–response curves were plotted to determine the IC_50_ and 95% confidence intervals for each cell line (Figure 2). As shown in Figure 3, *hMLH1*-deficient cell lines due to either epigenetic silencing (RKO) or biallelic mutation (HCT-116) were the most sensitive to CPT-11 in comparison to the MMR-proficient HT-29, which had an IC_50_ four- to nine-fold higher (MSI-H *vs* MSI-L/MSS, *P*<0.001). In addition, both cell lines presenting hypermethylation of *hMLH1* had a similar IC_50_ showing consistency in our results. However, the *hMSH6*-deficient cell line, HCT-15, displayed sensitivity to CPT-11 closer to the MSS cell line than the rest of MSI cell lines, suggesting a minor role of *hMSH6* deficiency in the sensitivity to CPT-11.

Regarding the mutational status of DSB repair genes and response to CPT-11, we have observed that HCT-116 and RKO, which harboured mutations in both *MRE11* and *RAD50*, were the most sensitive cell lines. On the contrary, HT-29 was wild type for both genes and was most resistant to CPT-11. Noteworthy, HCT-15 was heterozygous for the poly(T) mutation in *MRE11* and wild type for *RAD50* and was less sensitive to CPT-11 than HCT-116 and RKO. Therefore, the presence of mutations in DSB repair genes may explain the differences in sensitivity between MSI and MSS cells, whereas the number of affected DSB repair genes may account for different levels of sensitivity to CPT-11.

### Cell cycle analysis after treatment with different concentrations of CPT-11

**Figure 4:**
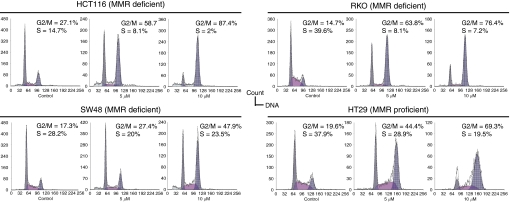
Exposure to CPT-11 for 48 h results in G2/M arrest. CRC cell lines were treated with 5 *μ*M, 10 *μ*M CPT-11, and supplemented medium (control). Cell cycle analyses were performed by FACS.

We did perform cell cycle analysis based on DNA content after exposure to two different concentrations of CPT-11 for 48 h. We selected 5 and 10 *μ*mol l^−1^ because the former is around the IC_25_ in the most sensitive cells (MMR deficient) and the latter is around the IC_50_ in the least sensitive (MMR proficient). Moreover, these concentrations are similar to peak plasma concentrations after CPT-11 infusion in human subjects (Mathijssen *et al*, 2001). As shown in Figure 4, the percentage of cells in S phase without treatment was 14.7–39.6%, and in G2 phase was 14.7–27.1%. Treatment with CPT-11 decreased the percentage of cells in S phase and elicited an arrest in the G2/M phase of cell cycle. When we compared the changes in the percentage of cells in S, G1 and G2/M between control and cells treated with 10 *μ*mol l^−1^ of CPT-11, RKO, and HCT-116 showed the most striking changes followed by HT-29. SW-48 elicited the same changes in G1, G2/M, and S phases; however, differences in relative percentages were smaller than in the other three cell lines. Overall, a similar pattern of changes in the cell cycle was observed in all cell lines.

## Discussion

The aim of this study was to correlate the cytotoxicity of the TOP1 inhibitor, CPT-11, with the mutational status of MMR and DSB genes in CRC cell lines. Our experimental data show that three cell lines displaying MSI-H due to an *hMLH1* inactivation are four- to nine-fold more sensitive to CPT-11 than an MSS cell line and that this difference in sensitivity is apparently independent from the original cause of the *hMLH1* deficiency. Moreover, cell lines with either *hMLH1* promoter hypermethylation or biallelic mutation have similar IC_50_ values. Additionally, we noticed that the *hMSH6*-deficient cell line, HCT-15, has sensitivity to CPT-11 closer to the MSS cell line than to the other MSI cell lines. In addition to this, those cell lines doubly mutated in *MRE11* and *RAD50* were more sensitive to CPT-11 than those cells heterozygous for the poly(T) mutation in MRE11, or wild type for both genes. Cell cycle analyses showed G2/M arrest and decrease in the number of cells in the S phase. These results are in agreement with the ‘fork collision model’ and the S-phase specificity of CPT-11. Therefore, this work reinforces previous reports of higher sensitivity of MMR-deficient cell lines to CPT-11 and offers a potential explanation for it (Jacob *et al*, 2001; Magrini *et al*, 2002).

Several authors have demonstrated that MSI is a prognostic marker in patients with CRC (Gryfe *et al*, 2000; Popat *et al*, 2005). Additionally, the presence of MSI may have a predictive value of response to 5-FU and CPT-11 in CRC. One multicentre retrospective review of clinicopathological data (Benatti *et al*, 2005) as well as two retrospective analysis of clinical trials in the adjuvant setting (Watanabe *et al*, 2001; Ribic *et al*, 2003) have suggested that patients with MSI-H may have less sensitivity to 5-FU-based chemotherapy than those with MSI-L/MSS tumours. In accordance with this fact, insensitivity to 5-FU has been consistently proved in various preclinical models with MSI-H CRC cell lines (Carethers *et al*, 1999; Meyers *et al*, 2001, 2005).

However, sensitivity of CPT-11 in this subset of tumours is not so clearly defined at both the preclinical and the clinical levels. Two independent research groups have reported resistance to camptothecins in MMR-deficient cell lines (Hausner *et al*, 1999; Fedier *et al*, 2001). In contrast with these reports, we and others (Jacob *et al*, 2001; Magrini *et al*, 2002) have demonstrated higher sensitivity to CPT-11 in MMR-deficient human CRC cell lines than in MMR-proficient cells and G2/M arrest after exposure to CPT-11. Likewise, the body of evidence supporting the sensitivity to CPT-11 in MSI-H CRC at the clinical level is limited. A retrospective analysis of 73 metastatic CRC patients who received second-line CPT-11-based chemotherapy showed a higher response rate in the group of tumours displaying MSI-H compared with the MSI-L/MSS group (57.1 *vs* 10.8%, *P-*value*=*0.009) (Fallik *et al*, 2003). Bertagnolli *et al* (2006) analysed the differences in disease-free survival in 482 patients enroled in the CALGB 89803 adjuvant study according to the microsatellite status . A total of 1264 patients with resected stage III colon cancer were randomized to receive adjuvant 5-FU-based treatment with or without CPT-11. Sixteen percent of patients had MSI-H tumours. Regarding the population receiving CPT-11 in addition to 5-FU, those patients bearing MSI-H tumours presented a higher disease-free survival than those patients with MSI-L/MSS tumours. Despite this, improvement in survival did not achieve statistical significance, although there was a clear trend for a prolongation in disease-free survival (long rank=0.18). Several reasons may explain the lack of significant differences found in this study. First, the results may not be mature enough due to the limited follow-up of the patients (mean follow-up=3.8 years). Second, the retrospective nature of this analysis along with the restriction to only one-third of the patients included in the clinical study may either be generating an uncontrolled bias or limiting the power to detect differences. Finally, adjuvant CPT-11-based chemotherapy has globally failed to demonstrate a significant improvement in terms of progression-free survival in stage III CRC in three different studies (van Cutsem *et al*, 2005; Ychou *et al*, 2005; Saltz *et al*, 2007), which suggests that factors other than MSI status play an important role as prognostic factors in the adjuvant setting.

The current evidence in the treatment of advanced CRC patients shows that multiagent chemotherapy incorporating either oxaliplatin (de Gramont *et al*, 2000; Giacchetti *et al*, 2000) or CPT-11 (Douillard *et al*, 2000; Saltz *et al*, 2000) onto a backbone of fluoropyrimidines is superior to traditional regimens of fluoropyrimidines alone (Kelly and Goldberg, 2005). However, the available randomised studies have failed to demonstrate that a first-line oxaliplatin combined with infusional 5-FU schedule, or a CPT-11 with infusional 5-FU schedule, was better than the other for the global population of patients with advanced CRC (Giacchetti *et al*, 2000; Tournigand *et al*, 2004). Further exploration on the role of microsatellite status to select the most appropriate drug combination for the first-line treatment of metastatic CRC may be considered in the design of future clinical trials.

One of the questions that still remains partially unanswered is the exact mechanism why MSI-H cells are more sensitive to camptothecins than MSS cells. Several studies have demonstrated that deficiency in the MMR genes leads to secondary mutations in microsatellite tracts as those present in the monucleotide repeats regions of the principal caretaker genes implicated in the repair of DSB, such as the Mre11/Nbs1/Rad50 protein complex (Giannini *et al*, 2004; Koh *et al*, 2005). These observations have been extended to tumour series from sporadic and familial MSI-H CRC cases (Miquel *et al*, 2007). We have suggested that cell lines harbouring mutations in both *MRE11* and *RAD50* were the most sensitive to CPT-11. Previous reports showed that mutations in these genes are also associated with increased response to *γ*-irradiation, another type of DNA-damaging agent causing DSB (Koh *et al*, 2005). In addition, the preferential cytotoxic effect showed by another DNA-damaging agent, Bleomycin, in MSI-H cell lines might be explained by the same genetic defect (Li *et al*, 2004). Still, further functional studies should be carried out to elucidate the relevance of this complex in the chemosensitivity to CPT-11 and other DNA-damaging agents.

In conclusion, our study reinforces previous preclinical results suggesting an increased sensitivity to CPT-11 in MSI-H CRC cell lines. This chemosensitivity is explained by an impairment of the mechanism to repair DSB caused by DNA-damaging agents secondary to the microsatelite instability. In addition, we have demonstrated that no differences in sensitivity exist between cell lines with inactivation of the Mlh-1 function secondary to biallelic mutations and those with epigenetic silencing of the promoter. These results together with the available clinical information support the design of more clinical trials with CPT-11-based chemotherapy regimens in the adjuvant and advanced settings according to a tailored approach that combines clinical characteristics and the MSI status as a predictive factor of response to chemotherapy.

## Figures and Tables

**Table 1:** MS, *hMLH1* promoter methylation, MMR genes status, and mutations in mononucleotide repeats of *MRE11* and *RAD50* alleles in cell lines

**Cell line**	**MS status**	***hMLH1* promoter methylation**	***hMLH1***	***hMSH2***	***hMSH6***	***RAD50* poly(A)9 exon 13**	***MRE11* poly(T)11 intron 4**
HCT-116	MSI-H	−	mut	wt	wt	−1/wt	−2/−1
HCT-15	MSI-L	−	wt	wt	mut	wt/wt	−2/wt
SW-48	MSI-H	+	wt	wt	wt	wt/wt	−1/−1
RKO	MSI-H	+	wt	wt	wt	−1/wt	−2/−1
HT-29	MSS	−	wt	wt	wt	wt/wt	wt/wt

MS=microsatellite; MSI-H=high-frequency microsatellite instability; MSI-L=low-frequency microsatellite instability; MSS=microsatellite stability; mut=mutant; wt=wild type; −=negative; +=positive.

## References

[bib1] Aaltonen LA, Salovaara R, Kristo P, Canzian F, Hemminki A, Peltomaki P, Chadwick RB, Kaariainen H, Eskelinen M, Jarvinen H, Mecklin JP, de la Chapelle A (1998) Incidence of hereditary nonpolyposis colorectal cancer and the feasibility of molecular screening for the disease. N Engl J Med 338: 1481–1487 9593786 10.1056/NEJM199805213382101

[bib2] Benatti P, Gafa R, Barana D, Marino M, Scarselli A, Pedroni M, Maestri I, Guerzoni L, Roncucci L, Menigatti M, Roncari B, Maffei S, Rossi G, Ponti G, Santini A, Losi L, Di Gregorio C, Oliani C, Ponz de Leon M, Lanza G (2005) Microsatellite instability and colorectal cancer prognosis. Clin Cancer Res 11: 8332–8340 16322293 10.1158/1078-0432.CCR-05-1030

[bib3] Bertagnolli MM, Compton CC, Niedzwiecki D, Warren RS, Jewell S, Bailey GP, Mayer RJ, Goldberg R, Saltz L, Redston M (2006) Microsatellite instability predicts improved response to adjuvant therapy with irinotecan, 5-fluorouracil and leucovorin in stage III colon cancer. J Clin Oncol (Meeting Abstracts) 24: 10003–1000 10.1200/JCO.2008.18.2071 PMC2668707 19273709

[bib4] Carethers JM, Chauhan DP, Fink D, Nebel S, Bresalier RS, Howell SB, Boland CR (1999) Mismatch repair proficiency and *in vitro* response to 5-fluorouracil. Gastroenterology 117: 123–131 10381918 10.1016/s0016-5085(99)70558-5 PMC4343206

[bib5] de Gramont A, Figer A, Seymour M, Homerin M, Hmissi A, Cassidy J, Boni C, Cortes-Funes H, Cervantes A, Freyer G, Papamichael D, Le Bail N, Louvet C, Hendler D, de Braud F, Wilson C, Morvan F, Bonetti A (2000) Leucovorin and fluorouracil with or without oxaliplatin as first-line treatment in advanced colorectal cancer. J Clin Oncol 18: 2938–2947 10944126 10.1200/JCO.2000.18.16.2938

[bib6] Douillard JY, Cunningham D, Roth AD, Navarro M, James RD, Karasek P, Jandik P, Iveson T, Carmichael J, Alakl M, Gruia G, Awad L, Rougier P (2000) Irinotecan combined with fluorouracil compared with fluorouracil alone as first-line treatment for metastatic colorectal cancer: a multicentre randomised trial. Lancet 355: 1041–1047 10744089 10.1016/s0140-6736(00)02034-1

[bib7] Fallik D, Borrini F, Boige V, Viguier J, Jacob S, Miquel C, Sabourin JC, Ducreux M, Praz F (2003) Microsatellite instability is a predictive factor of the tumor response to irinotecan in patients with advanced colorectal cancer. Cancer Res 63: 5738–5744 14522894

[bib8] Fedier A, Fink D (2004) Mutations in DNA mismatch repair genes: implications for DNA damage signaling and drug sensitivity (review). Int J Oncol 24: 1039–1047 15010846

[bib9] Fedier A, Schwarz VA, Walt H, Carpini RD, Haller U, Fink D (2001) Resistance to topoisomerase poisons due to loss of DNA mismatch repair. Int J Cancer 93: 571–576 11477562 10.1002/ijc.1356

[bib10] Giacchetti S, Perpoint B, Zidani R, Le Bail N, Faggiuolo R, Focan C, Chollet P, Llory JF, Letourneau Y, Coudert B, Bertheaut-Cvitkovic F, Larregain-Fournier D, Le Rol A, Walter S, Adam R, Misset JL, Levi F (2000) Phase III multicenter randomized trial of oxaliplatin added to chronomodulated fluorouracil-leucovorin as first-line treatment of metastatic colorectal cancer. J Clin Oncol 18: 136–13 10623704 10.1200/JCO.2000.18.1.136

[bib11] Giannini G, Rinaldi C, Ristori E, Ambrosini MI, Cerignoli F, Viel A, Bidoli E, Berni S, D'Amati G, Scambia G, Frati L, Screpanti I, Gulino A (2004) Mutations of an intronic repeat induce impaired MRE11 expression in primary human cancer with microsatellite instability. Oncogene 23: 2640–2647 15048091 10.1038/sj.onc.1207409

[bib12] Giannini G, Ristori E, Cerignoli F, Rinaldi C, Zani M, Viel A, Ottini L, Crescenzi M, Martinotti S, Bignami M, Frati L, Screpanti I, Gulino A (2002) Human MRE11 is inactivated in mismatch repair-deficient cancers. EMBO Rep 3: 248–254 11850399 10.1093/embo-reports/kvf044 PMC1084012

[bib13] Giardiello FM, Brensinger JD, Petersen GM (2001) AGA technical review on hereditary colorectal cancer and genetic testing. Gastroenterology 121: 198–213 11438509 10.1053/gast.2001.25581

[bib14] Gryfe R, Kim H, Hsieh ET, Aronson MD, Holowaty EJ, Bull SB, Redston M, Gallinger S (2000) Tumor microsatellite instability and clinical outcome in young patients with colorectal cancer. N Engl J Med 342: 69–77 10631274 10.1056/NEJM200001133420201

[bib15] Hausner P, Venzon DJ, Grogan L, Kirsch IR (1999) The ‘comparative growth assay’: examining the interplay of anti-cancer agents with cells carrying single gene alterations. Neoplasia 1: 356–367 10935491 10.1038/sj.neo.7900047 PMC1508098

[bib16] Herman JG, Umar A, Polyak K, Graff JR, Ahuja N, Issa JP, Markowitz S, Willson JK, Hamilton SR, Kinzler KW, Kane MF, Kolodner RD, Vogelstein B, Kunkel TA, Baylin SB (1998) Incidence and functional consequences of hMLH1 promoter hypermethylation in colorectal carcinoma. Proc Natl Acad Sci USA 95: 6870–6875 9618505 10.1073/pnas.95.12.6870 PMC22665

[bib17] Jacob S, Aguado M, Fallik D, Praz F (2001) The role of the DNA mismatch repair system in the cytotoxicity of the topoisomerase inhibitors camptothecin and etoposide to human colorectal cancer cells. Cancer Res 61: 6555–6562 11522654

[bib18] Jiricny J (2006) The multifaceted mismatch-repair system. Nat Rev Mol Cell Biol 7: 335–346 16612326 10.1038/nrm1907

[bib19] Kelly H, Goldberg RM (2005) Systemic therapy for metastatic colorectal cancer: current options, current evidence. J Clin Oncol 23: 4553–4560 16002847 10.1200/JCO.2005.17.749

[bib20] Kim NG, Choi YR, Baek MJ, Kim YH, Kang H, Kim NK, Min JS, Kim H (2001) Frameshift mutations at coding mononucleotide repeats of the hRAD50 gene in gastrointestinal carcinomas with microsatellite instability. Cancer Res 61: 36–38 11196187 10.1186/bcr362 PMC3300545

[bib21] Koh KH, Kang HJ, Li LS, Kim NG, You KT, Yang E, Kim H, Kim HJ, Yun CO, Kim KS, Kim H (2005) Impaired nonhomologous end-joining in mismatch repair-deficient colon carcinomas. Lab Invest 85: 1130–1138 16025146 10.1038/labinvest.3700315

[bib22] Kunkel TA (2004) DNA replication fidelity. J Biol Chem 279: 16895–16898 14988392 10.1074/jbc.R400006200

[bib23] Li HR, Shagisultanova EI, Yamashita K, Piao Z, Perucho M, Malkhosyan SR (2004) Hypersensitivity of tumor cell lines with microsatellite instability to DNA double strand break producing chemotherapeutic agent bleomycin. Cancer Res 64: 4760–4767 15256444 10.1158/0008-5472.CAN-04-0975

[bib24] Magrini R, Bhonde MR, Hanski ML, Notter M, Scherubl H, Boland CR, Zeitz M, Hanski C (2002) Cellular effects of CPT-11 on colon carcinoma cells: dependence on p53 and hMLH1 status. Int J Cancer 101: 23–31 12209584 10.1002/ijc.10565

[bib25] Mathijssen RH, van Alphen RJ, Verweij J, Loos WJ, Nooter K, Stoter G, Sparreboom A (2001) Clinical pharmacokinetics and metabolism of irinotecan (CPT-11). Clin Cancer Res 7: 2182–2194 11489791

[bib26] Meyers M, Wagner MW, Hwang HS, Kinsella TJ, Boothman DA (2001) Role of the hMLH1 DNA mismatch repair protein in fluoropyrimidine-mediated cell death and cell cycle responses. Cancer Res 61: 5193–5201 11431359

[bib27] Meyers M, Wagner MW, Mazurek A, Schmutte C, Fishel R, Boothman DA (2005) DNA mismatch repair-dependent response to fluoropyrimidine-generated damage. J Biol Chem 280: 5516–5526 15611052 10.1074/jbc.M412105200

[bib28] Miquel C, Jacob S, Grandjouan S, Aime A, Viguier J, Sabourin JC, Sarasin A, Duval A, Praz F (2007) Frequent alteration of DNA damage signalling and repair pathways in human colorectal cancers with microsatellite instability. Oncogene 26: 5919–5926 17384679 10.1038/sj.onc.1210419

[bib29] Pommier Y (2006) Topoisomerase I inhibitors: camptothecins and beyond. Nat Rev Cancer 6: 789–802 16990856 10.1038/nrc1977

[bib30] Popat S, Hubner R, Houlston RS (2005) Systematic review of microsatellite instability and colorectal cancer prognosis. J Clin Oncol 23: 609–618 15659508 10.1200/JCO.2005.01.086

[bib31] Ribic CM, Sargent DJ, Moore MJ, Thibodeau SN, French AJ, Goldberg RM, Hamilton SR, Laurent-Puig P, Gryfe R, Shepherd LE, Tu D, Redston M, Gallinger S (2003) Tumor microsatellite-instability status as a predictor of benefit from fluorouracil-based adjuvant chemotherapy for colon cancer. N Engl J Med 349: 247–257 12867608 10.1056/NEJMoa022289 PMC3584639

[bib32] Salovaara R, Loukola A, Kristo P, Kaariainen H, Ahtola H, Eskelinen M, Harkonen N, Julkunen R, Kangas E, Ojala S, Tulikoura J, Valkamo E, Jarvinen H, Mecklin JP, Aaltonen LA, de la Chapelle A (2000) Population-based molecular detection of hereditary nonpolyposis colorectal cancer. J Clin Oncol 18: 2193–2200 10829038 10.1200/JCO.2000.18.11.2193

[bib33] Saltz LB, Cox JV, Blanke C, Rosen LS, Fehrenbacher L, Moore MJ, Maroun JA, Ackland SP, Locker PK, Pirotta N, Elfring GL, Miller LL (2000) Irinotecan plus fluorouracil and leucovorin for metastatic colorectal cancer. Irinotecan Study Group. N Engl J Med 343: 905–914 11006366 10.1056/NEJM200009283431302

[bib34] Saltz LB, Niedzwiecki D, Hollis D, Goldberg RM, Hantel A, Thomas JP, Fields AL, Mayer RJ (2007) Irinotecan fluorouracil plus leucovorin is not superior to fluorouracil plus leucovorin alone as adjuvant treatment for stage III colon cancer: results of CALGB 89803. J Clin Oncol 25: 3456–3461 17687149 10.1200/JCO.2007.11.2144

[bib35] Suter CM, Norrie M, Ku SL, Cheong KF, Tomlinson I, Ward RL (2003) CpG island methylation is a common finding in colorectal cancer cell lines. Br J Cancer 88: 413–419 12569385 10.1038/sj.bjc.6600699 PMC2747532

[bib36] Tournigand C, Andre T, Achille E, Lledo G, Flesh M, Mery-Mignard D, Quinaux E, Couteau C, Buyse M, Ganem G, Landi B, Colin P, Louvet C, de Gramont A (2004) FOLFIRI followed by FOLFOX6 or the reverse sequence in advanced colorectal cancer: a randomized GERCOR study. J Clin Oncol 22: 229–237 14657227 10.1200/JCO.2004.05.113

[bib37] van Cutsem E, Labianca R, Hossfeld D, Bodoky G, Roth A, Aranda E, Nordlinger B, Assadourian S, Wang K, Cunningham D (2005) Randomized phase III trial comparing infused irinotecan/5-fluorouracil (5-FU)/folinic acid (IF) *vs* 5-FU/FA (F) in stage III colon cancer patients (pts). (PETACC 3). J Clin Oncol (Part 1) 23(16): (Suppl) 3S–3S

[bib38] Watanabe T, Wu T-T, Catalano PJ, Ueki T, Satriano R, Haller DG, Benson AB, Hamilton SR (2001) Molecular predictors of survival after adjuvant chemotherapy for colon cancer. N Engl J Med 344: 1196–1206 11309634 10.1056/NEJM200104193441603 PMC3584633

[bib39] Ychou M, Raoul JL, Douillard JY, Bugat R, Mineur L, Viret F, Becouarn Y, Bouche O, Jacob JH, Gourgou-Bourgade S, for the GIGotF, the F (2005) A phase III randomized trial of LV5FU2+CPT-11 vs. LV5FU2 alone in adjuvant high risk colon cancer (FNCLCC Accord02/FFCD9802). J Clin Oncol (Meeting Abstracts) 23: 3502–350

